# Risk of Early-Onset Neonatal Group B Streptococcal Disease With Maternal Colonization Worldwide: Systematic Review and Meta-analyses

**DOI:** 10.1093/cid/cix655

**Published:** 2017-11-06

**Authors:** Neal J Russell, Anna C Seale, Catherine O’Sullivan, Kirsty Le Doare, Paul T Heath, Joy E Lawn, Linda Bartlett, Clare Cutland, Michael Gravett, Margaret Ip, Shabir A Madhi, Craig E Rubens, Samir K Saha, Stephanie Schrag, Ajoke Sobanjo-ter Meulen, Johan Vekemans, Carol J Baker

**Affiliations:** 1 Maternal, Adolescent, Reproductive and Child Health Centre, London School of Hygiene & Tropical Medicine, United Kingdom;; 2 King’s College London, United Kingdom;; 3 College of Health and Medical Sciences, Haramaya University, Dire Dawa, Ethiopia;; 4 Paediatric Infectious Diseases Research Group, St George’s, University of London, United Kingdom;; 5 Centre for International Child Health, Imperial College London, United Kingdom;; 6 Department of International Health, Johns Hopkins Bloomberg School of Public Health, Baltimore, Maryland;; 7 Medical Research Council: Respiratory and Meningeal Pathogens Research Unit, and Department of Science and Technology/National Research Foundation: Vaccine Preventable Diseases, University of the Witwatersrand, Faculty of Health Sciences, Johannesburg, South Africa;; 8 Global Alliance to Prevent Prematurity and Stillbirth, Seattle, Washington;; 9 Department of Obstetrics and Gynecology, University of Washington, Seattle;; 10 Department of Microbiology, Faculty of Medicine, Chinese University of Hong Kong;; 11 National Institute for Communicable Diseases, National Health Laboratory Service, Johannesburg, South Africa;; 12 Department of Global Health, University of Washington, Seattle;; 13 Bangladesh Institute of Child Health, Dhaka;; 14 National Center for Immunization and Respiratory Diseases, Centers for Disease Control and Prevention, Atlanta, Georgia;; 15 Bill & Melinda Gates Foundation, Seattle, Washington;; 16 World Health Organization, Geneva, Switzerland; and; 17 Departments of Pediatrics and Molecular Virology and Microbiology, Baylor College of Medicine, Houston, Texas

**Keywords:** group B *Streptococcus*, *Streptococcus agalactiae*, vertical transmission, risk, neonatal sepsis

## Abstract

**Background:**

Early-onset group B streptococcal disease (EOGBS) occurs in neonates (days 0–6) born to pregnant women who are rectovaginally colonized with group B *Streptococcus* (GBS), but the risk of EOGBS from vertical transmission has not been systematically reviewed. This article, the seventh in a series on the burden of GBS disease, aims to estimate this risk and how it varies with coverage of intrapartum antibiotic prophylaxis (IAP), used to reduce the incidence of EOGBS.

**Methods:**

We conducted systematic reviews (Pubmed/Medline, Embase, Latin American and Caribbean Health Sciences Literature (LILACS), World Health Organization Library Information System [WHOLIS], and Scopus) and sought unpublished data from investigator groups on maternal GBS colonization and neonatal outcomes. We included articles with ≥200 GBS colonized pregnant women that reported IAP coverage. We did meta-analyses to determine pooled estimates of risk of EOGBS, and examined the association in risk of EOGBS with IAP coverage.

**Results:**

We identified 30 articles including 20328 GBS-colonized pregnant women for inclusion. The risk of EOGBS in settings without an IAP policy was 1.1% (95% confidence interval [CI], .6%–1.5%). As IAP increased, the risk of EOGBS decreased, with a linear association. Based on linear regression, the risk of EOGBS in settings with 80% IAP coverage was predicted to be 0.3% (95% CI, 0–.9).

**Conclusions:**

The risk of EOGBS among GBS-colonized pregnant women, from this first systematic review, is consistent with previous estimates from single studies (1%–2%). Increasing IAP coverage was linearly associated with decreased risk of EOGBS disease.

Maternal colonization with group B *Streptococcus* (GBS; *Streptococcus agalactiae*) is the most important risk factor for early-onset (0–6 days) invasive neonatal GBS disease (EOGBS). However, the risk of EOGBS in newborns born to GBS-colonized pregnant women has not previously been systematically reviewed and quantified. The first and most frequently referenced study is from 1973, where 1 infant among 46 pregnant women with vaginal GBS colonization developed EOGBS [1]—that is, around 2% risk. However, this was before intrapartum antibiotic prophylaxis (IAP) became established in high-income contexts.

Since the 1970s and 1980s [1–8], several observational studies and randomized controlled trials have demonstrated that IAP reduces the risk of EOGBS [9–17], using either microbiological screening (rectovaginal colonization) [16, 18] or clinical risk factors for EOGBS, such as preterm labor (<37 weeks), prolonged rupture of membranes (PROM) (>18 hours), maternal fever (≥38.0°C [100.4°F]), or suspicion of chorioamnionitis [18–21]. The risk of EOGBS disease may therefore vary, according to maternal GBS colonization prevalence, IAP policy, and effectiveness of IAP implementation.

This article, assessing the risk of neonatal disease in pregnant women colonized with GBS, is part of a supplement estimating the burden of GBS disease in pregnant women, stillbirths, and infants, which is important in terms of public health policy, particularly vaccine development (Figure 1), as outlined elsewhere [22]. The supplement includes systematic reviews and meta-analyses on GBS colonization and maternal and birth adverse outcomes associated with GBS [23–30], which form input parameters to a compartmental model to estimate the global burden of GBS [31].

**Figure 1.**
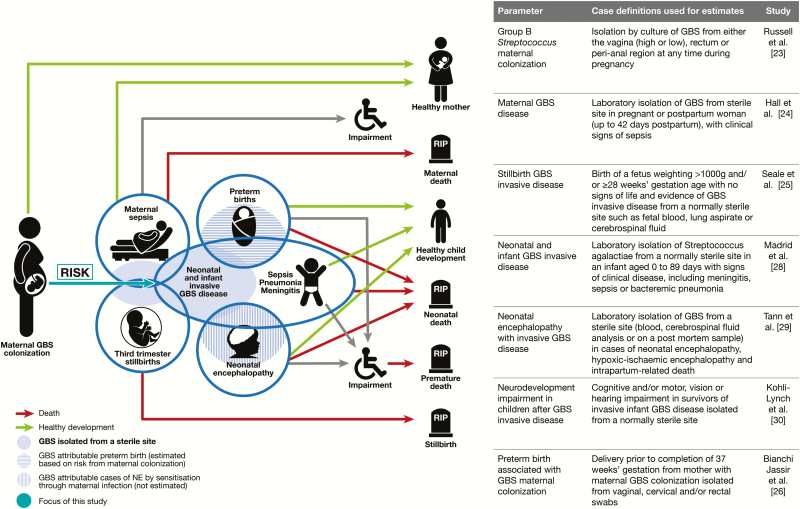
Risk of early onset neonatal disease in the disease schema for group B *Streptococcus*, as described by Lawn et al [22]. Abbreviations: GBS, group B *Streptococcus*; NE, neonatal encephalopathy.

The specific objectives of this paper are as follows:

1. To provide a comprehensive and systematic literature review and meta-analyses to assess the following parameters: (*i*) risk of EOGBS in settings without an IAP policy, (*ii*) risk of EOGBS at varying levels of IAP implementation (using a microbiological screening policy);
2. To assess the data for possible use for estimating the burden of EOGBS disease;
3. To evaluate the gaps in the data and recommend what should be done to improve the data on risk of EOGBS.

## METHODS

This article is part of a protocol entitled “Systematic estimates of the global burden of GBS in pregnant women, stillbirths and infants,” submitted for ethical approval to the London School of Hygiene & Tropical Medicine (reference number 11966) and approved on 30 November 2016.

### Definitions

Maternal GBS colonization was defined as isolation by culture of GBS from either the vagina (high or low), rectum, or perianal region during pregnancy. EOGBS was defined as GBS disease confirmed by microbiological culture of blood or cerebrospinal fluid (CSF) taken on days 0–6 [28]. We assumed that blood or CSF samples were obtained for a clinical indication. We excluded cases of “probable” GBS sepsis, where clinical or laboratory signs of infection were accompanied only by neonatal GBS colonization, and cases of clinically suspected pneumonia with GBS detected in tracheal aspirates, or urinary tract infections. Intrapartum antibiotic prophylaxis (IAP) was defined as intravenous antibiotics given at any time during labor for the prevention of EOGBS in GBS-colonized pregnant women. Coverage of IAP refers to the proportion of women who received IAP, regardless of the timing of administration. Studies were categorized as having a policy of IAP for GBS colonization if they aimed to provide IAP to all colonized pregnant women regardless of risk factors.

### Data Searches and Inputs

We identified data through systematic review of the published literature and through development of an investigator group of clinicians, researchers, and relevant professional institutions worldwide. For this article, all articles from a review of maternal GBS colonization [23] were reviewed for inclusion here. In addition, we searched reference lists of clinical trials [32, 33], and related systematic reviews [21, 34, 35] (Supplementary Table 1). There were no date or language restrictions. Articles were screened by 2 authors (N. R. and C. O.), both of whom independently assessed the studies for quality and risk of bias, and a third author’s opinion (A. S.) was requested in cases of differences of opinion.

Articles were included if they described a cohort of pregnant women with vaginal or rectovaginal GBS colonization, including newborn disease outcomes, and described use of IAP, including the proportion of pregnant women who received it (if any policy). Studies where women were not systematically screened for GBS colonization, but were provided with IAP based on clinical risk factors with unknown GBS colonization status, were not included. To reduce selection bias in studies with very small cohorts of pregnant women colonized with GBS, which could overestimate the risk of GBS disease through preferential reporting, articles were included if they reported outcomes from at least 200 pregnant women colonized with GBS. This was based on the 1%–2% risk of EOGBS previously reported, and the estimated number of women among whom there would be expected to be at least 1 case [1].

Studies were assessed for potential bias as reported in Supplementary Table 2. Articles were excluded if there was evidence of recruitment bias, such as studies where rectovaginal sampling was in response to clinical risk (which may overestimate disease risk) [19, 21].

We used random-effects meta-analyses to estimate the risk of EOGBS using the DerSimonian and Laird method [36]. We examined the relationship between IAP coverage and risk of EOGBS with linear regression.

Sensitivity analyses were done to explore bias in studies that did not include reporting on clinical risk factors for EOGBS. These analyses included:

1. Excluding studies that did not report presence or absence of any clinical risk factors;
2. Excluding studies without information on gestational age;
3. Excluding studies without reporting of PROM;
4. Excluding studies without reporting of maternal fever.

These sensitivity analyses were applied to studies regardless of IAP policy and then to those with and without IAP for GBS colonization separately.

## RESULTS

### Study Selection

**Figure 2.**
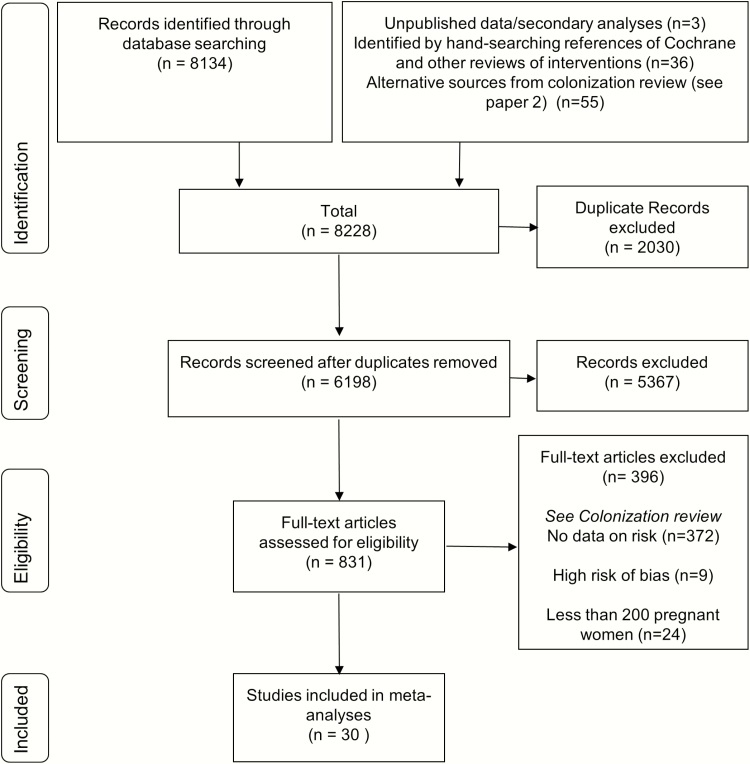
Data search and included studies for risk of early-onset neonatal invasive group B streptococcal disease in the presence of maternal colonization.

From a total of 6128 articles identified through the search on maternal colonization [23] and references lists of relevant reviews, we identified 30 articles that met the inclusion criteria, 14 of which included cohorts of pregnant women without a policy of providing IAP to all women with GBS colonization (Figure 2).

### Study Characteristics

**Figure 3.**
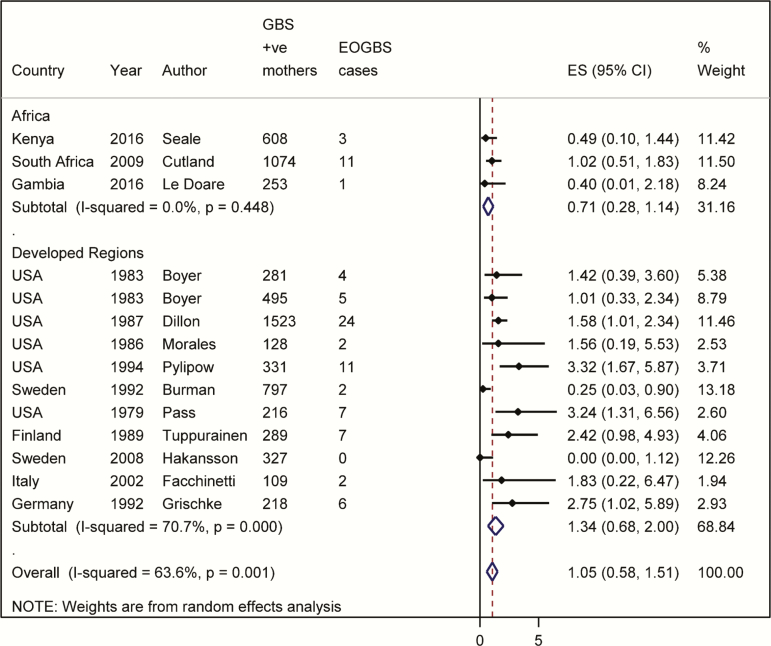
Meta-analysis of risk of early-onset disease without intrapartum antibiotic prophylaxis for group B *Streptococcus* (GBS) colonization. (Including 6649 GBS-colonized pregnant women and 85 early-onset GBS cases.) Abbreviations: CI, confidence interval; EOGBS, early-onset group B *Streptococcus*; ES, estimate; GBS, group B *Streptococcus*.

The majority of studies were observational (25/30), with 5 of 30 randomized controlled trials (of IAP or vaginal chlorhexidine aiming to reduce neonatal sepsis). Eligible articles included 20328 pregnant women colonized with GBS and 101 cases of EOGBS. Nine articles were from North America and 15 were from Europe, with 3 studies from Asia and 3 studies from Africa (The Gambia, Kenya, and South Africa; Figure 3). (See Supplementary Table 3 for study characteristics.) Of the included studies, 7 of 30 did not report the prevalence of clinical risk factors for EOGBS at delivery, and could therefore be subject to bias. Among studies reporting the prevalence of clinical risk factors, the prevalence of prolonged rupture of membranes (defined by most studies as >18 hours, one study as >24 hours) was 8% (9 studies), maternal fever (≥38.0°C) was 3% (6 studies), and prematurity (<37 weeks) was 5% (11 studies). A number of studies did not directly report on risk factors but reported proxy measures such as median birth weights (as a proxy for gestation).

### Outputs From Meta-analyses and Linear Regression

**Figure 4.**
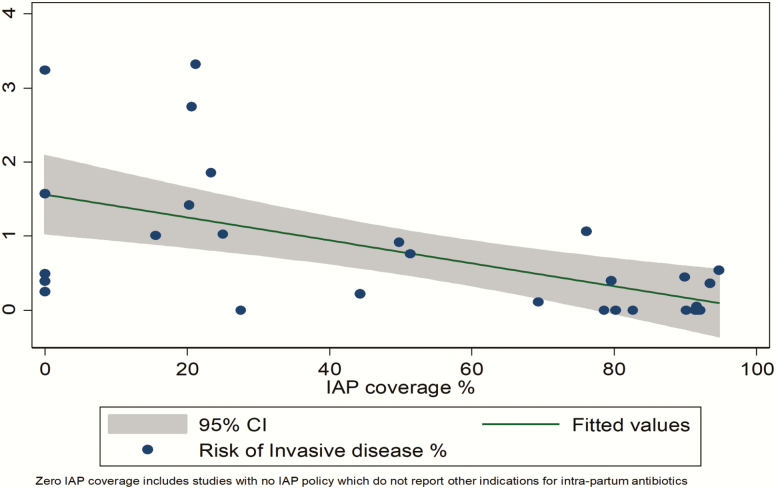
Risk of early-onset disease with varying intrapartum antibiotic prophylaxis coverage of group B *Streptococcus* (GBS)–colonized pregnant women. (Including 20328 GBS-positive pregnant women and 101 early-onset GBS cases.) Abbreviations: CI, confidence interval; IAP, intrapartum antibiotic prophylaxis.

In settings without a policy of providing IAP for GBS colonization, the risk of EOGBS in newborns of GBS colonized mothers was 1.1% (95% confidence interval [CI], .6%–1.5%) (Figure 3). Among the studies in this review where there was a policy of providing IAP for GBS colonization (including women who received IAP, as well those who missed IAP), the overall risk of EOGBS was much lower (0.03% [95% CI, 0–.07%]; Table 1 and Supplementary Figure 1), with a mean IAP coverage of 75%. When all studies were included, regardless of IAP policy, with increasing IAP coverage the risk of EOGBS decreased. Figure 4 shows IAP coverage against risk of EOGBS. This graph (linear regression line) can be used to estimate the risk of EOGBS based on different estimates of IAP coverage. Table 2 shows the varying expected risk of EOGBS with different coverage levels of IAP based on the linear association. For example, with coverage of IAP of 80%, the risk of EOGBS would be expected to be 0.3% (95% CI, 0–0.9%). Note that where “no coverage” is reported, this does not imply no antibiotics during labor, as antibiotics may have been administered for other indications.

**Table 1.** Summary of Risk of Early-Onset Group B *Streptococcus* by Intrapartum Antibiotic Prophylaxis Policy^a^

IAP Policy	No. of GBS- Colonized Mothers	No. of Early- Onset GBS Cases	Pooled Estimates (Worldwide)
No IAP policy	6649	85	1.1 (95% CI, .6–1.5)
IAP policy^b^ (varying coverage)	13348	16	0.03 (95% CI, 0–.07)

Abbreviations: CI, confidence interval; GBS, group B *Streptococcus*; IAP, intrapartum antibiotic prophylaxis.

^a^See Meta-analyses in the Supplementary Materials.

^b^Not including randomized controlled trials.

**Table 2.** Relationship Between Coverage of Intrapartum Antibiotic Prophylaxis and Risk of Invasive Early-Onset Group B Streptococcal (GBS) Disease From Cohorts of GBS-Positive Mothers From Linear Regression Model

Setting	Estimated Coverage	Risk (95% CI)	Risk Reduction (95% CI)
High coverage of microbiological screening-based policy (eg, US)	80%^a^	0.3% (0–.9%)	79.2% (45.5%–113%)
Microbiological screening-based policy with limited implementation	40%	0.9% (.4%–1.5%)	40% (6%–73%)
Risk-based strategy with high implementation, and ad hoc screening	60%^b^	0.6% (.1%–1.2%)	59% (26%–93%)
Risk-based strategy with high implementation	50%^b^	0.8% (.3%–1.3%)	50% (16%–83%)

Abbreviations: CI, confidence interval; US, United States.

^a^Based on US data on estimated coverage of GBS-positive pregnant women with screening and intrapartum antibiotic prophylaxis [56]

^b^Theoretical estimated coverages based on data that approximately 40% (or more) of newborns with early onset are born to pregnant women with no risk factors [19].

In the context of a policy of IAP for GBS colonization, studies did not consistently report the timing of administration of IAP, so analysis of the varying risk of invasive disease with the timing of the first antibiotic dose before delivery was not possible [37]).

Multiple sensitivity analyses were done to explore potential bias from the lack of reporting of clinical risk factors for EOGBS. These were as follows:

### Excluding Studies That Did Not Report Presence or Absence of Any Clinical Risk Factors

The risk of EOGBS did not differ significantly between studies that reported risk factors and those that did not, as shown by overlapping confidence intervals (Supplementary Table 4). Excluding studies that did not report any risk factor, the risk of EOGBS without IAP for GBS colonization was 0.9% (95% CI, .4%–1.4%). Including all studies regardless of IAP policy, overall risk was also not significantly different (0.3 [95% CI, .1–.4] in all vs 0.3 [95% CI, .1–.5] if reporting a risk factor).

### Excluding Studies Without Information on Gestational Age

The risk without IAP for GBS colonization among studies reporting gestational age of newborns was 0.9 (95% CI, .2–1.5).

### Excluding Studies Without Reporting of Prolonged Rupture of Membranes

The risk without IAP for GBS colonization among studies reporting prevalence of PROM was 0.8 (95% CI, .5–1.5).

### Excluding Studies Without Reporting of Maternal Fever

The risk without IAP for GBS colonization among studies reporting maternal fever was comparable to the primary analysis (1.4 [95% CI, .4–2.3]).

The outputs of these analyses, as well as the same analyses but including studies with IAP policies, and all studies, are summarized in Supplementary Table 4 and Supplementary Figures 2–6). These outputs were also comparable to the primary analysis.

## DISCUSSION

The risk of EOGBS was 1.1% (95% CI, .7%–1.6%) for newborns born to women colonized with GBS in pregnancy without a policy of providing IAP for positive GBS screening. As IAP coverage increased the risk of EOGBS decreased, with a linear relationship. This clear association allows assessment of risk of EOGBS in a population of GBS-colonized pregnant women, based on expected coverage of IAP.

This is the most comprehensive review to date of the risk of EOGBS disease in newborns born to pregnant women colonized with GBS. These results are consistent with previous studies [1, 38–41], but provide more robust estimates of the risk of EOGBS among pregnant women colonized with GBS and, importantly, how this varies with and without IAP. The inclusion of data from both high- and low-income contexts means the estimated risks are generalizable, and support estimates modeling disease burden where there are different IAP policies and coverage of IAP [27].

Some studies could have been biased because risk factors for EOGBS (prematurity, prolonged rupture of membranes ≥18 hours and maternal fever ≥38.0°C) were not reported. However in the case of prematurity, one of the most important risk factors for EOGBS [21], sensitivity analyses did not provide any evidence that the risk of EOGBS when including studies reporting gestational age differed from the primary analysis (0.9% [95% CI, .2%–1.5%] vs 1.1% [95% CI, .6%–1.5%]). Nevertheless, the prevalence of prematurity of 5% among the studies which reported proportion of preterm births, compared to a global estimate of preterm birth of 11.1% [42], suggested that preterm newborns may have been under-represented. In addition, most preterm neonates included were late preterm (35–36 weeks) or moderate preterm (≥32 weeks), because swabs for GBS screening are not routinely collected before 35 weeks’ gestation, creating a moderate selection bias. As most (84%) preterm deliveries occur after 32 weeks [43], and the majority of EOGBS occur in term newborns [18, 44–47], the degree of bias is likely to be modest. Underestimation of risk may also occur due to misclassification of the exposure. Maternal GBS colonization varies during pregnancy, and women detected as GBS colonized very early in pregnancy may no longer be colonized at delivery, but their newborns would be included as exposed, lowering the overall risk estimate.

Other known clinical risk factors for EOGBS disease, prolonged rupture of membranes (≥18 hours) or maternal fever (>38°C), were not frequently reported. However, the prevalence of both of these risk factors seems low [48, 49], and in many study settings fever was likely to result in antibiotic treatment. Both of these factors would likely lead to underestimation of the risk of EOGBS disease.

Significant underestimation of risk may also have been through inadequate case ascertainment, which is limited by the sensitivity of blood cultures. Furthermore, use of IAP may sterilize blood cultures without reducing clinical disease to the same extent. Indeed “probable” cases of EOGBS, where clinical signs of sepsis are associated with GBS colonization in newborns without other positive bacteriology, may represent a much higher incidence of disease than that based on positive blood cultures alone [50, 51]. Such cases are difficult to quantify, however, and were not included in this review. Case ascertainment is also reduced if newborns are not adequately followed up for the full 0- to 6-day early-onset period, but as the majority of EOGBS cases occur in the first 24 hours after birth, this reduces the possible underestimation [28].

Although less likely, overestimation is possible, as the majority of studies included were in hospital settings and could select for a higher risk group of women. Another source of overestimation, but applying only to a minority of studies (4 studies) included, was the use of insensitive microbiological methods (lack of selective enrichment) to detect GBS maternal colonization. This could overrepresent women with high density of GBS colonization, and thus increased risk of vertical transmission to their newborns [21].

There are likely other factors modifying the risk of EOGBS in the presence of maternal GBS colonization, leading to changing risk in different settings. These could be genetic, especially relating to ethnicity, but this was insufficiently described to permit further analyses. Serotypes and sequence type clonal complexes colonizing mothers may also be important, but sufficient paired data linking maternal colonizing serotypes with newborn invasive disease were not available to estimate any varying risk. Comorbidities may also be important; recent studies have suggested a higher risk of GBS disease in human immunodeficiency virus (HIV)–exposed as well as HIV-infected newborns (despite similar colonization prevalence), although this appears to have a greater effect on late-onset disease [46, 52].

This review included studies from 4 continents, but the majority of studies were from high-income contexts (United States or Europe). Applying a risk from high-income contexts to low- and middle-income contexts, where access to hospital care is limited, may underestimate disease as there may not be antibiotic treatment available, even in cases of clinically suspected maternal sepsis. There are other factors that may vary across settings, such as the proportion of births by elective cesarean delivery. Although emergency cesarean delivery in labor after ruptured membranes may not significantly change the risk of EOGBS with a GBS-colonized mother (risk will vary and may be higher depending on the indications for the procedure [53]), *elective* cesarean delivery before the onset of labor or rupture of membranes is associated with a much lower risk of EOGBS [54], which was not possible to quantify in this review. Therefore, settings with high rates of elective cesarean delivery before labor may have a lower risk of EOGBS than described here.

Importantly, this review should not be interpreted as implying no risk of EOGBS disease in newborns of pregnant women who test negative for GBS, as there may be false-negative results, and women may become colonized after screening and before delivery. In the context of high coverage of microbiological screening and IAP, a significant proportion of newborns with EOGBS disease are born to pregnant women who tested negative (or were not tested) for GBS colonization [41, 55].

Overall, our study shows the risk of EOGBS disease in GBS colonized pregnant women is at least 1 in 100, which is reduced with increasing IAP coverage based on microbiological screening. The risk is likely underestimated and will lead to a conservative minimum estimate of the burden of GBS disease in newborns in a compartmental model (Table 3).

**Table 3.** Key Findings and Implications

What’s new about this? • This is the first systematically derived estimate of risk of EOGBS disease worldwide, in the context of varying intrapartum antibiotic prophylaxis among pregnant women colonized with GBS.
What was the main finding? • Risk of EOGBS in newborns of pregnant women colonized with GBS is at least 1.1% without a policy of IAP for maternal GBS colonization. The risk decreased as coverage of IAP increases.
How can the data be improved? • More studies linking maternal and newborn data in different geographies, particularly Asia, are needed. Including serotype and MLST subtypes would help to explore differences in disease risk.
What does it mean for policy and programs? • This review provides a robust, but minimum, estimate of the risk of EOGBS given the population prevalence of maternal GBS colonization and estimated IAP coverage.

Abbreviations: EOGBS, early-onset group B *Streptococcus*; GBS, group B *Streptococcus*; IAP, intrapartum antibiotic prophylaxis; MLST, multilocus sequence typing.

## Supplementary Data

Supplementary materials are available at *Clinical Infectious Diseases* online. Consisting of data provided by the authors to benefit the reader, the posted materials are not copyedited and are the sole responsibility of the authors, so questions or comments should be addressed to the corresponding author.

## Supplementary Material

Supplement-material

## Notes

***Author contributions.*** The concept of the estimates and the technical oversight of the series were led by J. E. L. and A. C. S. The reviews, analyses, and first draft of the manuscript were undertaken by N. R. with A. C. S. and C. J. B. Other specific contributions were made by C. O. S., K. L. D., P. T. H. The GBS Estimates Expert Advisory Group (L. B., C. C., M. G. G., M. I., S. A. M., C. E. R., S. K. S., S. S., A. S.-t. M., J. V.) contributed to the conceptual process throughout, notably on the disease schema and data inputs. All the authors reviewed and input to the manuscript.

***Acknowledgments.*** The authors thank Claudia da Silva for administrative assistance and Alegria Perez for coordinating author signatures.

***Financial support.*** This supplement was supported by a grant to the London School of Hygiene & Tropical Medicine from the Bill & Melinda Gates Foundation (Grant ID: OPP1131158).

***Supplement sponsorship.*** This article appears as part of the supplement “The Burden of Group B *Streptococcus* Worldwide for Pregnant Women, Stillbirths, and Children,” sponsored by the Bill & Melinda Gates Foundation and coordinated by the London School of Hygiene & Tropical Medicine.

***Potential conflicts of interest.*** Many contributors to this supplement have received funding for their research from foundations, especially the Bill & Melinda Gates Foundation, and several from Wellcome Trust, Medical Research Council UK, the Thrasher Foundation, the Meningitis Research Foundation, and one individual from the US National Institutes of Health. Members of the Expert Advisory Group received reimbursement for travel expenses to attend working meetings related to this series. A. S.-t. M. works for the Bill & Melinda Gates Foundation. C. J. B. has served as a member of the Presidential Advisory Committee for Seqirus Inc and of the CureVac Inc Scientific Advisory Committee, as well as undertaken consultancy work for Pfizer Inc. C. C. has received institutional compensation from Novartis for conducting GBS studies. P. T. H. has been a consultant to Novartis and Pfizer on GBS vaccines but received no funding for these activities. M. I. has undertaken sponsored research from Pfizer on pneumococcal disease in adults and from Belpharma Eumedica (Belgium) on temocillin antimicrobial susceptibility in Enterobacteriaceae. K. L. D. has received funding by the Bill & Melinda Gates Foundation to work on research on GBS serocorrelates of protection to inform vaccine trials, and travel expenses from Pfizer to attend a meeting on an investigator-led project on GBS. S. A. M. has collaborated on G. B. S. grants funded by GlaxoSmithKline and by Pfizer and received personal fees for being member of its advisory committee; he has also collaborated on a GBS grant funded by Minervax. All other authors report no potential conflicts of interest. All authors have submitted the ICMJE Form for Disclosure of Potential Conflicts of Interest. Conflicts that the editors consider relevant to the content of the manuscript have been disclosed.
