# Supplementary material for: Risk of Early-Onset Neonatal Group B Streptococcal Disease With Maternal Colonization Worldwide: Systematic Review and Meta-analyses
Source: Clin Infect Dis. 2017 Nov 6;65(Suppl 2):S152–9. doi: 10.1093/cid/cix655 (PMC5850448; doi:10.1093/cid/cix655)
Supplement: Supplement-material [file cix655_suppl_supplement-material.pdf]

The burden of Group B *Streptococcus* worldwide for pregnant women, stillbirths and children

Paper 7: Risk of early-onset neonatal group B *Streptococcus* disease with maternal colonization worldwide: systematic review and meta-analyses

Supplementary information

Contents

The burden of Group B *Streptococcus* worldwide for pregnant women, stillbirths and children 1

Paper 7: Risk of early-onset neonatal group B *Streptococcus* disease with maternal colonization worldwide: systematic review and meta-analyses 1

Supplementary information 1

Supplementary Table S1: Search strategy and sources of data 3

Supplementary Table S2: Assessment of potential for bias in studies 4

Supplementary Table S3: Characteristics of included studies 5

Supplementary Table S4: Summary of risk of EOD in different IAP policy contexts and in the presence of risk factors (sensitivity analyses) 7

Supplementary Figure S1: Meta-analysis of risk of EOD with a policy of IAP for GBS colonized mothers 8

Supplementary Figure S2: Meta-analysis of risk of EOGBS (all studies regardless of IAP policy) 9

Supplementary Figure S3: Meta-analysis of studies describing any risk factor (regardless of IAP policy) 10

Supplementary Figure S4: Meta-analysis of studies describing gestational age (regardless of IAP policy) 11

Supplementary Figure S5: Meta-analysis of studies describing prolonged rupture of membranes (regardless of IAP policy) 12

Supplementary Figure S6: Meta-analysis of studies describing maternal fever (regardless of IAP policy) 13

Supplementary Table S1: Search strategy and sources of data

- Maternal colonisation search (see Paper 2 of this Supplement)
  - No date restrictions *(including high income >10yrs old)*
  - Including all studies with a cohort of more than 200 GBS colonised mothers which report on neonatal outcomes with availability of blood and csf culture
- Recent systematic review of prevalence of early-onset neonatal infection among newborns of mothers with colonisation/infection (including but not limited to studies on GBS) (Chan et al 2015)
- Cochrane reviews
  - IAP for maternal colonisation (2014)
  - Chlorhexidine for prevention of neonatal GBS disease (2014)
- UK cost effectiveness review (Colbourn et al 2007)
- Reference lists of above
- *Review of risk of neonatal disease in US before CDC recommendations adopted (Benitz 1999) – papers included above*
- Unpublished, anonymised data provided by investigator group

Supplementary Table S2: Assessment of potential for bias in studies

Category	Risk grading	Example
**Selection bias**	High risk	Selection based on risk factors
	Moderate risk	Swabs taken after 35 weeks' gestation
	Low risk	Unselected representative sample
	Unclear risk	Selection inadequately described
**Misclassification of exposure**	High risk	PCR detection<br>Non vaginal swabs<br>Mothers tested for GBS colonization after diagnosis of newborn
	Moderate risk	Non selective culture methods
	Low risk	Selective enrichment
	Unclear risk	Methods of GBS detection in mothers not described
**Loss to follow-up of outcome**	High risk	Newborns of GBS colonized mothers not all followed up
	Moderate risk	Follow up < 3 days
	Low risk	Follow up 3-6 days
	Unclear risk	No description of length of follow up
**Overrepresentation of high risk mothers**	High risk	High prevalence of any risk factor (>30%)
	Moderate risk	Moderately high prevalence of a risk factor (10-30%)
	Low risk	Representative prevalence of risk factors
	Unclear risk	No description of risk factors
**Misclassification of outcome**	High risk	Culture diagnosis not available<br>EOD cases not from within the original cohort of GBS colonized mothers
	Moderate risk	Non-selective culture methods
	Low risk	Selective enrichment<br>Automated culture
	Unclear risk	Methods not described

Supplementary Table S3: Characteristics of included studies

Country	Author	Year	Design *	Exclusions	IAP policy for GBS colonization?	Intra-partum antibiotic** (%)	GBS colonized mothers (n)	EOGBS cases	Any risk factors	Risk of bias[#]	Direction of bias[##]
Qatar	Al taher[1]	2008	Obsv		Y	80	550	0	Y	Mod	Under
Italy	Berardi[2]	2014	Obsv	<35 wks, Planned elective c section, non-Ampicillin IAP	Y	92	499	0	Y	Mod	Under
Spain	Bosch Mestres[3]	1997	Obsv		Y	93	551	2	Y	Low	Unc
USA	Boyer[4]	1983	Obsv		N	20	281	4	N	Unc	Unc
USA	Boyer[5]	1983	Obsv		N	16	495	5	Y	Unc	Unc
Sweden	Burman[6]	1992	RCT	<37wks, planned elective c-section, multiple pregnancy, congenital abnormality, previous infant with GBS, antibiotics within 2 weeks of swab	N	0	797	2	Y	Mod	Under
Italy	Cantoni[7]	2013	Obsv	preterm	Y	83	3010	0	Y	Mod	Under
SouthAfrica	Cutland[8] Secondary Analysis	2009	RCT	Planned c-section, APH, Congenital Malformation, IUD, Age<15, chlorhex allergy, face, gen warts, full cervical dilatation	RCT	0	825	10	Y		
Spain	De Cueto[9]	1998	Obsv	none	Y	44	454	1	Y	Unc	Unc
Italy	Della Morte[10]	1996	Obsv	?	Y	76	376	4	Y	Unc	Unc
USA	Dillon[11]	1987	Obsv		N	0	1523	24	Y	Mod	Under
Italy	Facchinetti[12]	2002	RCT	<37 weeks, multiple pregnancy, ROM >6 hrs, C section	RCT	50	217	2	Y	Mod	Under
USA	Faro[13]	2010	Obsv		Y	95	552	3	Y	Mod	Under
Germany	Grischke[14]	1992	Obsv	?	N	21	218	6	N	Unc	Unc
Austria	Hafner[15]	1998	Obsv	< 33 weeks	Y	91	520	0	N	Mod	Under

Sweden	Hakansson[16]	2008	Obsv	none	N	28	327	0	Y	Low	Unc
Israel	Hashavya[17]	2011	Obsv		Y	92	3819	2	N	Mod	Unc
USA	Katz[18]	1994	Obsv		Y	92	237	0	Y	Low/ Mod	Unc /Under
Poland	Kociszewska-Najman[19]	2010	Obsv		Y	80	250	1	N	Low	Under
Gambia	Le Doare[20]	2016	Obsv	not planning to breastfeed, HIV positive	N		253	1	N	Mod	Under
Taiwan	Lin[21]	2011	Obsv		Y	90	447	2	Y	Mod	Under
USA	Lin[22]	2011	Obsv	<32 weeks	Y	69	904	1	Y	Mod	Under
USA	Morales[23]	1986	RCT	<36 weeks, planned elective c-section	RCT	51	263	2	Y	Mod	Under
USA	Pass[24]	1979	Obsv		N	0	216	7	Y	Unc	Unc
USA	Pylipow[25]	1994	Obsv		N	21	331	11	Y	Low/ mod	Under
Kenya	Seale[26] (secondary analysis)	2016	Obsv	none	N	0	608	3	Y	Mod	Under
Poland	Szymusik[27]	2014	Obsv	none	Y	79	220	0	Y	Mod	Under
France	Thibaudon Baveux[28]	2008	Obsv		Y	90	283	0	Y	Mod	Under
Finland	Tuppurainen[29]	1989	RCT	c-section	RCT	23	377	7	N	Mod	Over
Italy	Zuppa[30]	2014	Obsv	planned elective c-section	Y	92	676	0	Y	Mod	Under

*Design: Obsv=observational, RCT=Randomized controlled trial

**Includes any intravenous antibiotics, either in the context of a policy of IAP for GBS colonization, or as treatment of a suspected infection, or in response to risk factors.

#Risk of bias: Unc=Unclear, Mod=moderate

##Direction of bias: Under=Underestimate, Over=overestimate, Unc=Unclear

Supplementary Table S4: Summary of risk of EOD in different IAP policy contexts and in the presence of risk factors (sensitivity analyses)

Meta-analysis	Risk %	Risk if any risk factor described	Risk only if gestation described	Risk if PROM described	Risk if maternal fever described
All studies	0.26%(0.13-0.40)	0.32(0.14-0.50)	0.29(0.08-0.51)	0.32(0.0-0.65)	0.52(0.04-1.00)
No IAP policy	1.05 (0.58-1.51)	0.89 (0.41-1.37)	0.88 (0.23-1.53)	0.78 (0.48-1.49)	1.36 (0.43-2.29)
IAP policy (varying coverage)	0.03 (0-0.07)	0.03(0-0.07)	0.01(0-0.07)	0.04(0-0.24)	0.04(0-0.25)

Supplementary Figure S1: Meta-analysis of risk of EOD with a policy of IAP for GBS colonized mothers

Country	Year	Author	GBS +ve mothers	EOGBS cases	IAP coverage %	ES (95% CI)	% Weight
.							
Developed Regions							
Poland	2010	Kociszewska-Najman	250	1	80	0.40 (0.01, 2.21)	0.18
Poland	2014	Szymusik	220	0	79	0.00 (0.00, 1.66)	0.32
USA	1994	Katz	237	0	92	0.00 (0.00, 1.54)	0.37
USA	2011	Lin	904	1	69	0.11 (0.00, 0.61)	2.37
Austria	1998	Hafner	520	0	91	0.00 (0.00, 0.71)	1.78
Italy	2014	Berardi	499	0	92	0.00 (0.00, 0.74)	1.64
Spain	1998	De Cueto	454	1	44	0.22 (0.01, 1.22)	0.60
Spain	1997	Bosch Mestres	551	2	93	0.36 (0.04, 1.30)	0.56
Italy	1996	Della Morte	376	4	76	1.06 (0.29, 2.70)	0.15
Italy	2014	Zuppa	676	0	92	0.00 (0.00, 0.54)	3.00
Italy	2013	Cantoni	3010	0	83	0.00 (0.00, 0.12)	59.16
USA	2010	Faro	552	3	95	0.54 (0.11, 1.58)	0.41
France	2008	Thibaudon Baveux	283	0	90	0.00 (0.00, 1.30)	0.53
Subtotal (I-squared = 0.0%, p = 0.814)						0.01 (-0.04, 0.07)	71.06
.							
Asia							
Israel	2011	Hashavya	3819	2	92	0.05 (0.01, 0.19)	26.58
Qatar	2008	Al taher	550	0	80	0.00 (0.00, 0.67)	1.99
Taiwan	2011	Lin	447	2	90	0.45 (0.05, 1.61)	0.37
Subtotal (I-squared = 0.0%, p = 0.580)						0.05 (-0.03, 0.14)	28.94
.							
Overall (I-squared = 0.0%, p = 0.864)						0.03 (-0.02, 0.07)	100.00

NOTE: Weights are from random effects analysis

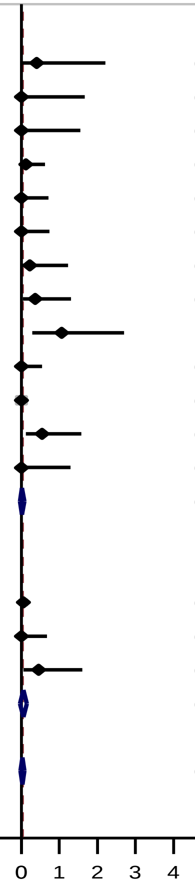

Supplementary Figure S2: Meta-analysis of risk of EOGBS (all studies regardless of IAP policy)

Country	Year	Author	GBS +ve mothers	EOGBS cases	ES (95% CI)	% Weight
Africa						
Kenya	2016	Seale	608	3	0.49 (0.10, 1.44)	2.94
South Africa	2009	Cutland	1074	11	1.02 (0.51, 1.83)	3.00
Gambia	2016	Le Doare	253	1	0.40 (0.01, 2.18)	1.33
Subtotal (I-squared = 0.0%, p = 0.448)					0.71 (0.28, 1.14)	7.27
.						
Developed Regions						
Poland	2010	Kociszewska-Najman	250	1	0.40 (0.01, 2.21)	1.30
Poland	2014	Szymusik	220	0	0.00 (0.00, 1.66)	2.09
USA	1983	Boyer	281	4	1.42 (0.39, 3.60)	0.65
USA	1983	Boyer	495	5	1.01 (0.33, 2.34)	1.52
USA	1987	Dillon	1523	24	1.58 (1.01, 2.34)	2.97
USA	1994	Katz	237	0	0.00 (0.00, 1.54)	2.35
USA	1986	Morales	263	2	0.76 (0.09, 2.72)	0.94
USA	1994	Pylipow	331	11	3.32 (1.67, 5.87)	0.39
USA	2011	Lin	904	1	0.11 (0.00, 0.61)	6.95
Sweden	1992	Burman	797	2	0.25 (0.03, 0.90)	5.04
USA	1979	Pass	216	7	3.24 (1.31, 6.56)	0.25
Finland	1989	Tuppurainen	377	7	1.86 (0.75, 3.79)	0.72
Sweden	2008	Hakansson	327	0	0.00 (0.00, 1.12)	3.73
Austria	1998	Hafner	520	0	0.00 (0.00, 0.71)	6.20
Italy	2014	Berardi	499	0	0.00 (0.00, 0.74)	5.97
Spain	1998	De Cueto	454	1	0.22 (0.01, 1.22)	3.35
Italy	2002	Facchinetti	217	2	0.92 (0.11, 3.29)	0.66
Spain	1997	Bosch Mestres	551	2	0.36 (0.04, 1.30)	3.18
Italy	1996	Della Morte	376	4	1.06 (0.29, 2.70)	1.10
Italy	2014	Zuppa	676	0	0.00 (0.00, 0.54)	7.53
Italy	2013	Cantoni	3010	0	0.00 (0.00, 0.12)	10.70
USA	2010	Faro	552	3	0.54 (0.11, 1.58)	2.54
Germany	1992	Grischke	218	6	2.75 (1.02, 5.89)	0.29
France	2008	Thibaudon Baveux	283	0	0.00 (0.00, 1.30)	3.06
Subtotal (I-squared = 64.1%, p = 0.000)					0.33 (0.14, 0.52)	73.50
.						
Asia						
Israel	2011	Hashavya	3819	2	0.05 (0.01, 0.19)	10.41
Qatar	2008	Al taher	550	0	0.00 (0.00, 0.67)	6.49
Taiwan	2011	Lin	447	2	0.45 (0.05, 1.61)	2.33
Subtotal (I-squared = 0.0%, p = 0.580)					0.05 (-0.03, 0.14)	19.24
.						
Overall (I-squared = 61.7%, p = 0.000)					0.26 (0.13, 0.40)	100.00

NOTE: Weights are from random effects analysis

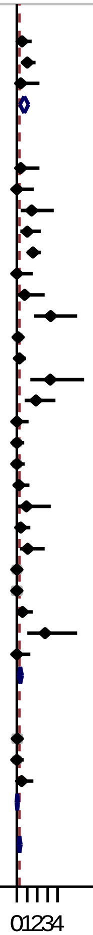

Supplementary Figure S3: Meta-analysis of studies describing any risk factor (regardless of IAP policy)

Country	Year	Author	GBS +ve mothers	EOGBS cases	ES (95% CI)	% Weight
Africa						
Kenya	2016	Seale	608	3	0.49 (0.10, 1.44)	4.19
South Africa	2009	Cutland	1074	11	1.02 (0.51, 1.83)	4.27
Subtotal (I-squared = 19.1%, p = 0.266)					0.76 (0.24, 1.28)	8.45
.						
Developed Regions						
Poland	2014	Szymusik	220	0	0.00 (0.00, 1.66)	3.16
USA	1983	Boyer	495	5	1.01 (0.33, 2.34)	2.39
USA	1987	Dillon	1523	24	1.58 (1.01, 2.34)	4.23
USA	1994	Katz	237	0	0.00 (0.00, 1.54)	3.49
USA	1986	Morales	263	2	0.76 (0.09, 2.72)	1.55
USA	1994	Pylipow	331	11	3.32 (1.67, 5.87)	0.67
USA	2011	Lin	904	1	0.11 (0.00, 0.61)	7.83
Sweden	1992	Burman	797	2	0.25 (0.03, 0.90)	6.31
USA	1979	Pass	216	7	3.24 (1.31, 6.56)	0.44
Sweden	2008	Hakansson	327	0	0.00 (0.00, 1.12)	5.06
Italy	2014	Berardi	499	0	0.00 (0.00, 0.74)	7.09
Spain	1998	De Cueto	454	1	0.22 (0.01, 1.22)	4.65
Italy	2002	Facchinetti	217	2	0.92 (0.11, 3.29)	1.12
Spain	1997	Bosch Mestres	551	2	0.36 (0.04, 1.30)	4.47
Italy	1996	Della Morte	376	4	1.06 (0.29, 2.70)	1.79
Italy	2014	Zuppa	676	0	0.00 (0.00, 0.54)	8.23
Italy	2013	Cantoni	3010	0	0.00 (0.00, 0.12)	10.07
USA	2010	Faro	552	3	0.54 (0.11, 1.58)	3.72
France	2008	Thibaudon Baveux	283	0	0.00 (0.00, 1.30)	4.33
Subtotal (I-squared = 64.4%, p = 0.000)					0.30 (0.10, 0.50)	80.59
.						
Asia						
Qatar	2008	Al taher	550	0	0.00 (0.00, 0.67)	7.49
Taiwan	2011	Lin	447	2	0.45 (0.05, 1.61)	3.46
Subtotal (I-squared = 7.1%, p = 0.299)					0.08 (-0.26, 0.42)	10.96
.						
Overall (I-squared = 64.5%, p = 0.000)					0.32 (0.14, 0.50)	100.00

NOTE: Weights are from random effects analysis

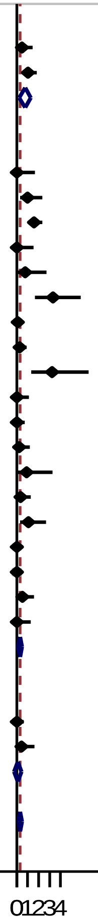

Supplementary Figure S4: Meta-analysis of studies describing gestational age (regardless of IAP policy)

Country	Year	Author	GBS +ve mothers	EOGBS cases	ES (95% CI)	% Weight
Developed Regions						
USA	1987	Dillon	1523	24	1.58 (1.01, 2.34)	6.02
USA	1994	Katz	237	0	0.00 (0.00, 1.54)	4.99
USA	1986	Morales	263	2	0.76 (0.09, 2.72)	2.26
USA	1994	Pylipow	331	11	3.32 (1.67, 5.87)	0.98
USA	2011	Lin	904	1	0.11 (0.00, 0.61)	10.85
Sweden	1992	Burman	797	2	0.25 (0.03, 0.90)	8.85
Sweden	2008	Hakansson	327	0	0.00 (0.00, 1.12)	7.16
Italy	2014	Berardi	499	0	0.00 (0.00, 0.74)	9.88
Spain	1997	Bosch Mestres	551	2	0.36 (0.04, 1.30)	6.35
Italy	2014	Zuppa	676	0	0.00 (0.00, 0.54)	11.37
Italy	2013	Cantoni	3010	0	0.00 (0.00, 0.12)	13.73
USA	2010	Faro	552	3	0.54 (0.11, 1.58)	5.32
France	2008	Thibaudon Baveux	283	0	0.00 (0.00, 1.30)	6.17
Subtotal (I-squared = 67.4%, p = 0.000)					0.23 (0.02, 0.44)	93.93
.						
Africa						
South Africa	2009	Cutland	1074	11	1.02 (0.51, 1.83)	6.07
Subtotal (I-squared = .%, p = .)					1.02 (0.37, 1.68)	6.07
.						
Overall (I-squared = 71.5%, p = 0.000)					0.29 (0.08, 0.51)	100.00

NOTE: Weights are from random effects analysis

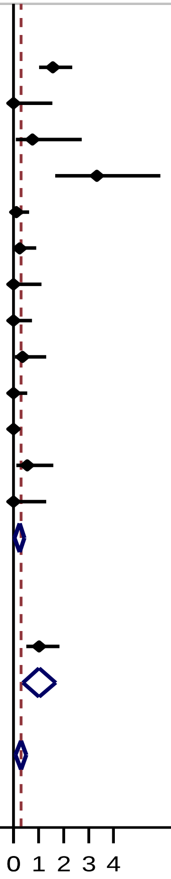

Supplementary Figure S5: Meta-analysis of studies describing prolonged rupture of membranes (regardless of IAP policy)

Country	Year	Author	GBS +ve mothers	EOGBS cases	ES (95% CI)	% Weight
Africa						
Kenya	2016	Seale	608	3	0.49 (0.10, 1.44)	11.41
South Africa	2009	Cutland	1074	11	1.02 (0.51, 1.83)	11.58
Subtotal (I-squared = 19.1%, p = 0.266)					0.76 (0.24, 1.28)	22.99
.						
Developed Regions						
USA	1994	Katz	237	0	0.00 (0.00, 1.54)	9.80
USA	1994	Pylipow	331	11	3.32 (1.67, 5.87)	2.15
Sweden	2008	Hakansson	327	0	0.00 (0.00, 1.12)	13.28
Italy	2014	Berardi	499	0	0.00 (0.00, 0.74)	17.16
Italy	2002	Facchinetti	217	2	0.92 (0.11, 3.29)	3.50
Spain	1997	Bosch Mestres	551	2	0.36 (0.04, 1.30)	12.02
Italy	2014	Zuppa	676	0	0.00 (0.00, 0.54)	19.08
Subtotal (I-squared = 48.5%, p = 0.070)					0.16 (-0.16, 0.47)	77.01
.						
Overall (I-squared = 60.3%, p = 0.010)					0.32 (-0.00, 0.65)	100.00

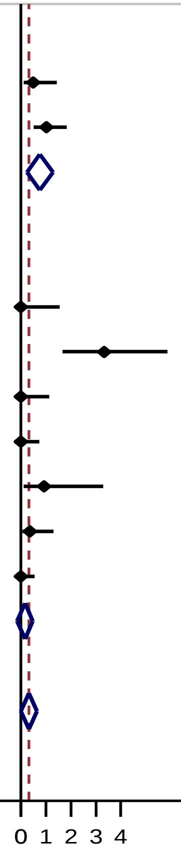

NOTE: Weights are from random effects analysis

Supplementary Figure S6: Meta-analysis of studies describing maternal fever (regardless of IAP policy)

Country	Year	Author	GBS +ve mothers	EOGBS cases	ES (95% CI)	% Weight
Developed Regions						
USA	1983	Boyer	495	5	1.01 (0.33, 2.34)	12.29
USA	1994	Pylipow	331	11	3.32 (1.67, 5.87)	4.40
Italy	2014	Berardi	499	0	0.00 (0.00, 0.74)	22.93
Spain	1997	Bosch Mestres	551	2	0.36 (0.04, 1.30)	18.22
Italy	2014	Zuppa	676	0	0.00 (0.00, 0.54)	24.41
Subtotal (I-squared = 70.7%, p = 0.008)					0.37 (-0.11, 0.85)	82.25
.						
Africa						
South Africa	2009	Cutland	1074	11	1.02 (0.51, 1.83)	17.75
Subtotal (I-squared = .%, p = .)					1.02 (0.37, 1.68)	17.75
.						
Overall (I-squared = 75.6%, p = 0.001)					0.52 (0.04, 1.00)	100.00

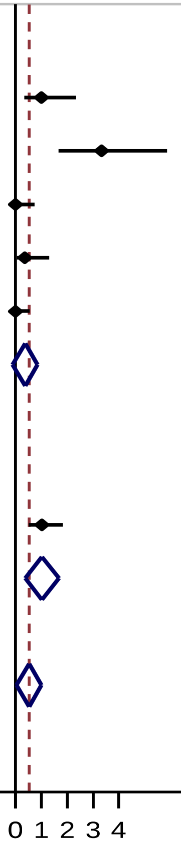

NOTE: Weights are from random effects analysis
